# Segregation of three resting-state brain networks predicts reappraisal success across the lifespan

**DOI:** 10.1093/scan/nsaf055

**Published:** 2025-05-21

**Authors:** Jordan E Pierce, Maital Neta

**Affiliations:** Department of Psychology & Center for Brain, Biology, and Behavior, University of Nebraska-Lincoln, Lincoln, NE 68588-0156, United States; Department of Psychology & Center for Brain, Biology, and Behavior, University of Nebraska-Lincoln, Lincoln, NE 68588-0156, United States

**Keywords:** cognitive reappraisal, resting-state functional connectivity, default mode, emotion regulation, network segregation

## Abstract

Cognitive reappraisal is a form of emotion regulation that involves the reinterpretation of stimuli to change one’s emotional state, often to reduce negative affect. Emotion regulation functional magnetic resonance imaging (fMRI) tasks generally yield increased activation in prefrontal cortex and, less consistently, decreased amygdala activation. Only a few studies, however, have examined how intrinsic brain organization, characterized via resting-state fMRI, relates to reappraisal, typically focusing on the same task-derived brain regions. Here, we administered an emotion regulation task where participants (*n* = 227, 6–80 years) viewed or downregulated responses to negative images, then completed a resting-state fMRI scan. We examined the functional connectivity in 300 whole-brain regions of interest comprising 13 functional networks. We found that the network segregation, or relative balance of within- and between-network connectivity, in the default mode network (DMN), dorsal attention network (DAN), and somatomotor-dorsal (SMd) network was associated with reappraisal success (controlling for age and movement). Specifically, greater connectivity within the DMN and DAN, lower connectivity within the SMd, and greater connectivity between the SMd and lateral SM networks predicted better reappraisal ability. These networks also partially overlapped with brain areas supporting emotion regulation and reactivity, suggesting that functional brain organization is a key factor in shaping emotion regulation across the lifespan.

## Introduction

Cognitive reappraisal is a form of emotion regulation that involves reinterpretation of an emotional stimulus in order to shift one’s emotional state, often to minimize negative feelings (Gross 2015, McRae 2016). Reappraisal is considered one of the more adaptive regulation strategies and can lead to long-term changes in emotion processing and better mental health outcomes (Gross and John 2003, Denny and Ochsner 2014, Denny et al. 2015, Kivity and Huppert 2016). Indeed, reappraisal is often utilized in clinical therapy to help reduce depression or anxiety symptoms (Goldin et al. 2012, Kazantzis et al. 2018), and can moderate the impact of stress on mood and well-being (Troy et al. 2010, Riepenhausen et al. 2022).

Reappraisal ability or success can be measured using laboratory tasks that compare emotion ratings or physiological responses during reappraisal with natural viewing conditions, such that effective regulation results in up- or down-regulation of emotional responses according to the desired goal. The neural correlates of reappraisal have been studied extensively using task-based functional magnetic resonance imaging (fMRI), showing heightened activity in various regions of the prefrontal cortex (PFC), as well as superior parietal and temporal cortex (Ochsner et al. 2012, Buhle et al. 2014, Frank et al. 2014, Helion et al. 2019, Steward et al. 2021, Bo et al. 2024). These regions are involved in various processes including attention, inhibition, cognitive control, semantic interpretations, and valuation (Ochsner et al. 2012, Denny et al. 2023), and may at least partially overlap with regions supporting emotion generation (Zhang et al. 2023a, Bo et al. 2024). While the aforementioned brain regions are more active during reappraisal than during baseline viewing conditions, other regions, such as the amygdala or insula, show the reverse pattern (Kanske et al. 2011, Buhle et al. 2014, Min et al. 2022).

In addition to examining the neural correlates of reappraisal using task-based fMRI, some studies have investigated how the organization of brain networks at rest contributes to reappraisal tendencies (i.e. self-report of habitual use) or reappraisal success (i.e. task-based performance). Resting-state brain organization reflects, in part, an individual’s history of functional coactivation of brain regions—experience with particular cognitive or affective functions can strengthen the connections between relevant network nodes (Van Den Heuvel and Pol 2010, Wig 2017). Individuals with stronger connectivity between certain regions at rest may therefore be better prepared to respond when asked to perform a reappraisal task that depends upon those brain networks. One study (Li et al. 2021) selected the inferior frontal gyrus (IFG) as a seed region of interest (ROI) and found that the resting-state functional connectivity (RSFC) between IFG and bilateral temporal and parietal cortex, as well as between dorsomedial PFC and cingulate cortex was related to reappraisal tendency. Another study (Morawetz et al. 2024) identified a set of ROIs (including IFG, supplementary motor area, middle temporal gyrus, and precuneus) based on their reappraisal task activation; they then estimated the effective connectivity in these ROIs during resting-state. Connections between frontal and temporal cortex predicted reappraisal success differentially for high and low intensity stimuli. Together, these previous reports indicate that the RSFC of widespread brain regions, including frontal, parietal, and temporal cortex, support various aspects of reappraisal.

These studies demonstrated a link between brain organization at rest and reappraisal, yet largely focused on task-based ROIs (Morawetz et al. 2017, 2024, Picó-Pérez et al. 2018, Li et al. 2021). While these regions certainly contribute to reappraisal ability, brain areas outside those specifically activated during task performance may also support one’s general preparation or aptitude for reappraisal-related processes. In the current work, rather than analysing only regions uniquely activated by reappraisal, we utilized a set of whole-brain ROIs encompassing multiple functional networks to examine the possible impact of brain organization on a broader scale. The current analysis investigated RSFC in terms of network segregation, which refers to the relative balance of within- and between-network connectivity (Chan et al. 2014, Wig 2017, Zhang et al. 2023b, Pierce et al. 2024). Brain networks should have high within-network connectivity to create a useful functional unit and sparse between-network connectivity to allow for the exchange of distinct information across networks (Sporns and Betzel 2016, Wig 2017). Optimized network segregation is necessary to allow for the efficient and flexible cognitions and behaviours that arise from the interactions of well-organized networks.

Prior work using this approach has identified an age-related decline in network segregation across the lifespan, with desegregation of higher-order association networks related to reduced long-term memory function (Chan et al. 2014) and symptoms of dementia (Zhang et al. 2023b). We have also recently demonstrated that segregation of the default mode network (DMN) is related to emotional ambiguity processing across the lifespan (Pierce et al. 2024). The myriad processes that contribute to reappraisal may similarly depend on higher-level organization across multiple networks to facilitate controlled emotional responses. For example, strong connectivity within a fronto-parietal network coupled with sparse connectivity between the fronto-parietal and reward networks may be necessary to accomplish the emotional appraisals and attentional control thereof that are required for effective reappraisal. Network segregation offers a single measure that represents how integrated or distinct these networks and their associated functions are.

Another important contributor to reappraisal success is age—studies have demonstrated that children and older adults differ in how effectively they implement emotion regulation (Tucker et al. 2012, Helion et al. 2019, Riediger and Bellingtier 2022). Changes in control networks across the lifespan may be one driving factor behind these differences, as the PFC is still developing in children and may exhibit atrophy in older adults (Helion et al. 2019). Specifically, there is evidence that children are worse at reappraisal and show weaker ventrolateral PFC activation compared to adolescents and young adults (McRae et al. 2012). Moreover, older adults may be worse at using reappraisal to decrease negative emotions, with weaker activity in some, but not all, PFC regions compared to younger adults (Winecoff et al. 2011, Opitz et al. 2012). These studies demonstrated age-based behavioural and task-based activation differences in reappraisal, yet previous work examining the relationship between reappraisal and RSFC has primarily utilized young adults. It is therefore unclear whether similar brain organization supports reappraisal across different stages of life or if unique neural mechanisms are engaged as the brain matures.

In the current study, participants across the lifespan (6–80 years old) completed a reappraisal fMRI task and a resting-state scan. Reappraisal success was operationalized as a decrease in negativity ratings when reappraising compared to viewing negative images. We aimed to identify age-related changes in reappraisal, predicting that reappraisal success would initially improve with age, then decrease in older adults. In addition, we aimed to determine whether reappraisal success could be predicted from resting-state brain organization, specifically network segregation, which was calculated from the RSFC of 13 functional networks. Critically, we predicted that segregation of control networks involved in reappraisal and affective networks involved in emotional reactivity would predict individual differences in reappraisal success. We also expected that age would modify these relationships, given the possibility that children and older adults may engage in different neural mechanisms during emotion regulation. Given the link between reappraisal and well-being noted earlier, the present work could help to identify brain regions that may be susceptible to dysfunction in individuals with mood disorders or psychopathology.

## Methods

### Participants

Three hundred and fifty-five participants were recruited from the Lincoln, Nebraska community and completed an initial pre-scanning session. Participants had to be right-handed, have no history of neurological disorder, no current use of a psychotropic medication, and no MRI contraindications. Of those who completed the first session, 44 were excluded for failing to meet MRI compatibility criteria, having poor screening data quality, or voluntarily opting out of the study. About one week later, 311 participants returned for the scanning session: of these, seven were unable to complete the resting-state scans, 21 were excluded for technical issues with behavioural responses or non-responses, and 56 were excluded for having an inadequate amount of resting-state data retained after motion censoring (described below). The final sample included 227 participants [M(SD)_age_ = 36.03 (22.14), range = 6–80; 138 female/89 male; 192 White, 17 more than one race, 9 Asian, and 9 Black; and 205 not Hispanic/Latino, 19 Hispanic/Latino, three no response]. All participants (and/or their legal guardians) confirmed their understanding of the procedures, provided written informed consent, and received compensation for their participation. All procedures were approved by the local institutional review board.

### Task design and procedure

In the emotion regulation task (see Pierce et al. 2022a for a full description), each trial began with an instruction screen lasting 2 s (‘Look’ or ‘Decrease’), followed by an emotional image from the International Affective Picture System (IAPS; Lang et al. 1997) lasting 7 s. For the look instruction trials, half of the images had a negative valence (‘Look Negative’) while the other half were neutral (‘Look Neutral’); participants were instructed to respond naturally and allow whatever feelings may arise. For the reappraise instruction trials (‘Decrease’), all images were negative and participants were instructed to cognitively reinterpret the content to feel less negative. Next, a rating screen appeared for 4 s where participants had to indicate their degree of negative emotion: ‘How bad do you feel?’ on a scale from 1 (not at all) to 5 (very bad). Finally, there was a ‘Rest’ screen that lasted 1, 2, or 3 s. There were 20 trials each of Look Negative, Look Neutral, and Decrease trials pseudo-randomly distributed throughout the task (60 total trials, all with unique images). Participants of all ages viewed the same set of images; parents of children under 17 years old screened and approved the images prior to their child’s participation, following recommendations from prior work (McRae et al. 2012, Abraham et al. 2024). Reappraisal success was calculated as the difference in ratings for ‘Look Negative’ minus ‘Decrease’ trials.

Participants completed a set of three practice trials (two ‘Decrease’ and one ‘Look Negative’ trial) and were asked to explain how they (re)appraised each scene to ensure task comprehension. Stimuli were presented using EPrime software (Psychological Software Tools, Inc., Pittsburgh, PA, United States) and response ratings were recorded via an MR-compatible button box. An anatomical scan was collected first, followed by a passive face viewing task (Petro et al. 2018, 2021), the emotion regulation task (see also Pierce et al. 2022a, 2022b), and resting-state scans (see also, Pierce et al. 2024), during which participants passively viewed a white crosshair on a black background. Adults typically completed one resting-state scan after the emotion regulation task, whereas children completed three shorter resting-state scans split before and after the emotion regulation task. Participant movement was monitored in real-time using FIRMM (Framewise Integrated Real-time MRI Monitoring, Dosenbach et al. 2017) and breaks were given or scan time extended as needed to acquire sufficient data with minimal head motion.

### Image acquisition

Data were collected on a Siemens 3 T Skyra scanner housed within the Center for Brain, Biology, and Behavior at the University of Nebraska-Lincoln. Structural images were collected using a T1-weighted Magnetization Prepared Rapid Gradient Echo (MP-RAGE) sequence (TR = 2.2 s, TE = 3.37 ms, slices = 192 interleaved, 1 mm isotropic voxel size, FOV = 256 mm, flip angle = 7^°^, total acquisition time = 5:07). Functional scans were collected using an EPI sequence: TR = 1.0 s, TE = 30 ms, slices = 51, voxel size = 2.5 mm isotropic, matrix = 84 × 84 mm, FOV = 210 mm^2^, flip angle = 60^°^, multiband factor = 3). The two emotion regulation task scans lasted 8:08 minutes each and the one to three resting-state scans lasted ∼15 minutes in total.

### Image preprocessing

Preprocessing was performed using fMRIPrep 23.2.1 (Esteban et al. 2019), which is based on Nipype 1.8.6 (Gorgolewski et al. 2011). The T1w image was corrected for intensity non-uniformity with N4BiasFieldCorrection (Tustison et al. 2010), distributed with ANTs 2.5.0 (Avants et al. 2008), and used as T1w-reference. The T1w-reference was then skull-stripped with a Nipype implementation of antsBrainExtraction.sh. Brain tissue segmentation of cerebrospinal fluid, white-matter, and gray-matter was performed using fast (FSL, Zhang et al. 2001). Brain surfaces were reconstructed using recon-all (FreeSurfer 7.3.2, Dale et al. 1999) and the brain mask was refined with a custom variation to reconcile ANTs-derived and FreeSurfer-derived segmentations of the cortical gray-matter of Mindboggle (Klein et al. 2017). Volume-based spatial normalization to standard space (MNI152NLin2009cAsym) was performed through nonlinear registration with antsRegistration. The following template was selected for spatial normalization and accessed with TemplateFlow (23.1.0, Ciric et al. 2022): ICBM 152 Nonlinear Asymmetrical template version 2009c (Fonov et al. 2009).

For each of the resting-state BOLD runs, a reference volume was generated, using a custom methodology of fMRIPrep, for use in head motion correction. Head-motion parameters with respect to the BOLD reference (transformation matrices, and six corresponding rotation/translation parameters) were estimated before any spatiotemporal filtering using mcflirt (FSL; Jenkinson et al. 2002). The BOLD reference was then co-registered to the T1w-reference using bbregister [FreeSurfer, (Greve and Fischl 2009)]. Co-registration was configured with six degrees of freedom.

### Functional connectivity processing

After preprocessing, resting-state functional data were analysed using in-house MATLAB scripts for functional connectivity processing (Power et al. 2012, Nielsen et al. 2019, Gratton et al. 2020) and included demeaning and detrending of each run, regression of nuisance variables (i.e. global signal, cerebrospinal and white matter nuisance masks derived from Freesurfer, and six rigid-body motion parameters, motion derivatives, and Volterra expansion of motion estimate; Friston et al. 1996), frame censoring and interpolation of data within runs, a temporal band-pass filter (0.009 Hz < *f *<* *0.08 Hz), and spatial smoothing (6 mm full width half maximum). Framewise displacement (FD) was calculated from realignment estimates and then low-pass filtered to remove high frequency noise (Gratton et al. 2020). Frames with greater than 0.2 mm FD were censored (removed) prior to analysis (Power et al. 2014, Nielsen et al. 2019). After censoring, data segments with less than five contiguous frames were removed, as were functional runs with fewer than 50 frames to ensure stability in the resting-state signal. Only participants with at least 800 remaining frames of resting data (13.3 minutes) were included, and the first 800 frames (after motion exclusions) were selected from each participant to minimize the effects of data quantity on network measures (Han et al. 2024). This cut-off was determined based on the distribution of retained frames in the current data (14–1311 frames, median = 923) to balance retention of participants (80.2%) and retention of data within retained participants (83.0%).

### Regions of interest

Resting-state functional connectivity time series were extracted from 300 whole-brain ROIs (Supplemental Table S1) with a 5 mm radius (Seitzman et al. 2020), then correlated to produce a correlation matrix, and normalized using a Fisher z-transform. This set of ROIs consists of 14 functional networks: Somatomotor-Dorsal (SMd), Somatomotor-Lateral, Cingulo-Opercular, Auditory, DMN, Parietal Medial, Visual, Fronto-Parietal, Salience, Ventral Attention, Dorsal Attention (DAN), Medial Temporal Lobe, Reward, and Unassigned. For network-level analyses, the unassigned ROIs were excluded, leaving 13 networks.

### Network segregation

Resting-state functional connectivity was analysed according to the methods described by Chan et al. (2014) for measuring within- and between-network functional connectivity, which were combined into the single metric of network segregation. Briefly, within-network connectivity was defined as the mean correlation (z-value) of all ROIs within a given network to each other, and between-network connectivity was defined as the mean correlation of all ROIs in a given network to all other ROIs in the brain or to all ROIs in each other network for network-level analyses. The segregation metric was defined as mean within- minus mean between-network correlation as a proportion of mean within-network correlation (scripts available at https://gitlab.com/wiglab/system-segregation-and-graph-tools) and represents the functional specialization of the network with respect to overall brain organization. As in prior work (Chan et al. 2014, 2018, Zhang et al. 2023b), all (unthresholded) positive correlations were included in the analysis, while all negative correlations were set to zero, given that global signal regression may introduce spurious negative correlations (Murphy et al. 2009).

### Linear models

Linear models were fit predicting reappraisal success (standardized) from segregation (controlled for FD), with standardized age (linear and quadratic effects) included as a covariate. The mean FD per participant was regressed out of each RSFC measure to further control for any effects of motion (segregation and FD: *r *=* −*.40, *P* < .001). *P*-values were corrected using the false discovery rate (FDR) for the overall model fits to control for multiple comparisons across the 13 networks. Based on the results of this primary analysis, follow-up analyses were conducted on the within- and between-network connectivity of the DMN, DAN, and SMd to further dissect the observed segregation effects. Given the wide age range in our sample, we also included interaction terms between age and within- and between-network connectivity. The results showed an effect of between-network connectivity only in the SMd and, therefore, connectivity between the SMd and each of the 12 other networks was next entered into a model predicting reappraisal, along with age. Augmented backward elimination (Dunkler et al. 2014) was used for stepwise selection of variables based on the Akaike information criterion (AIC), as in prior work (Pierce et al. 2024). Model stability was assessed using bootstrapped resampling (*n *= 1000; Heinze et al. 2018) to provide additional information about the distribution, variance and bias of the coefficient estimate for each predictor. Network segregation and linear model analyses were conducted in R Statistical Software, version 4.3.1 (R Core Team 2023).

### Comparison with task-based activation

To investigate the degree to which the RSFC results corresponded to activation during the emotion regulation task, the three networks identified by the network segregation analysis were spatially compared to a group-level task activation mask. Task data were analysed using the AFNI software package (Cox 1996, 2012). Preprocessing included de-spiking of time series outliers, slice timing correction, alignment of functional volumes to each other and the individual anatomical image, standardization to the Talairach atlas space (Talairach and Tournoux 1988), smoothing with a 6 mm FWHM kernel, and scaling of each voxel to a mean of 100. Next the data were entered into a general linear model with regressors for each trial type (Decrease, Look Negative, Look Neutral) and regressors of no interest consisting of polynomials for each run (four terms) and the six motion parameters estimated during alignment (x, y, z shift/rotation). Individual beta maps were entered into a whole brain one sample *t*-test to obtain group maps for the Decrease—Look Negative contrast (and its inverse), with a voxel-wise threshold of *P* < .005 and a cluster-wise threshold of *a* < .05. Next, the ROIs comprising the DMN, DAN, and SMd networks were transformed into Talairach space for comparison with the group map. As an initial visual comparison, the network ROIs were overlaid onto the thresholded group maps for the Decrease > Look Negative (reappraisal activity) and Look Negative > Decrease (emotional reactivity) contrasts. Finally, to quantify the task activity in these ROIs, the beta values for the unthresholded Decrease > Look Negative contrast were extracted from each participant and averaged for each of the ROIs in the three networks. A *t*-test vs. 0 was then conducted on the average contrast values within each network.

## Results

### Behavioural effects

**Figure 1.**
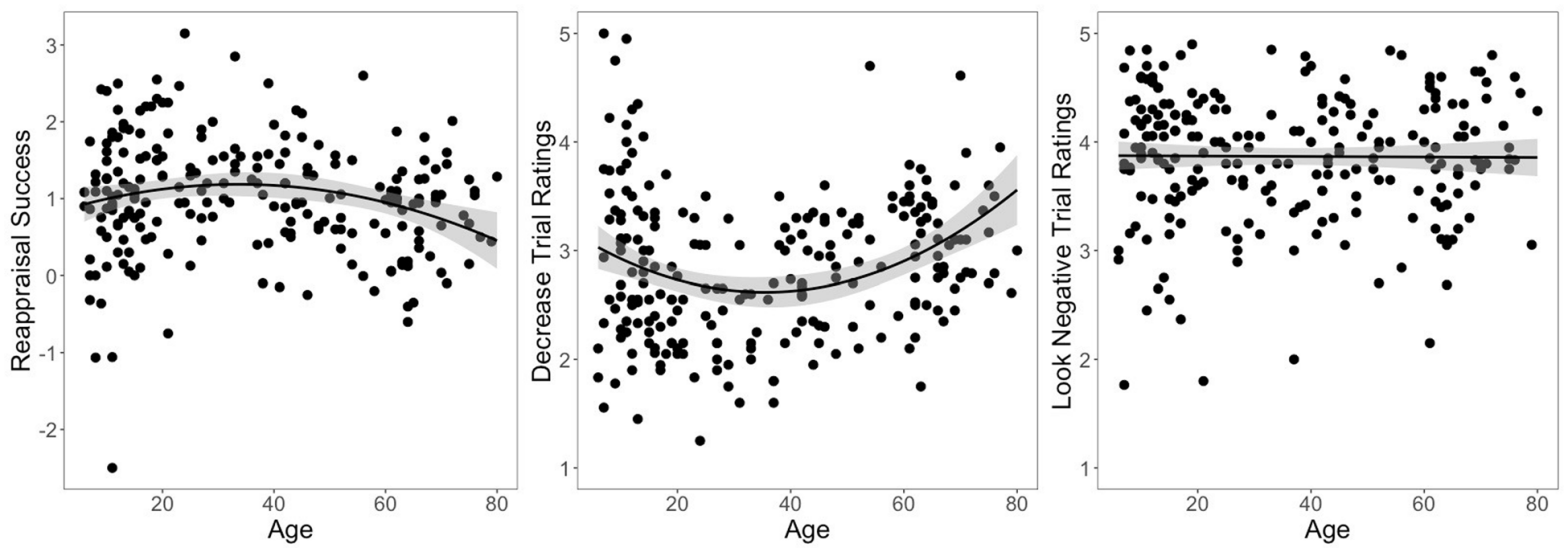
Relationship between age and reappraisal success (left), Decrease trial ratings (centre), and Look Negative trial ratings (right). Young adults showed the best reappraisal performance, with the largest reduction in ratings of negative feelings on Decrease trials relative to Look Negative trials (which showed no effect of age). Positive reappraisal success values indicate successful reappraisal while negative values indicate higher ratings of negative feelings during reappraisal compared to baseline viewing; Decrease and Look Negative trial ratings ranged from 1 (not at all negative) to 5 (very negative).

Reappraisal success was defined as the difference in ratings (1 to 5 scale) between Look Negative and Decrease trials, with positive values indicating a successful reduction of negative feelings during regulation; the mean reappraisal success was 1.02 (SD = 0.75). A linear model predicting reappraisal success from age (*F*(2, 224) = 4.90, *P* = .008, *R*^2^ = .04) showed a significant quadratic effect (*b *=* −*0.17, *t *=* −*2.59, *P* = .01), with the highest reappraisal success in young adults and slightly worse performance in older adults (Fig. 1). This effect was driven primarily by differences in Decrease trial ratings, which also showed a quadratic effect with age (*F*(2, 224) = 10.33, *P* < .001, *R*^2^ = .08), while Look Negative trials did not significantly differ with age (*F*(2, 224) < 1, *P* = .442, *R*^2^ = .007).

### Network segregation and reappraisal success

**Figure 2.**
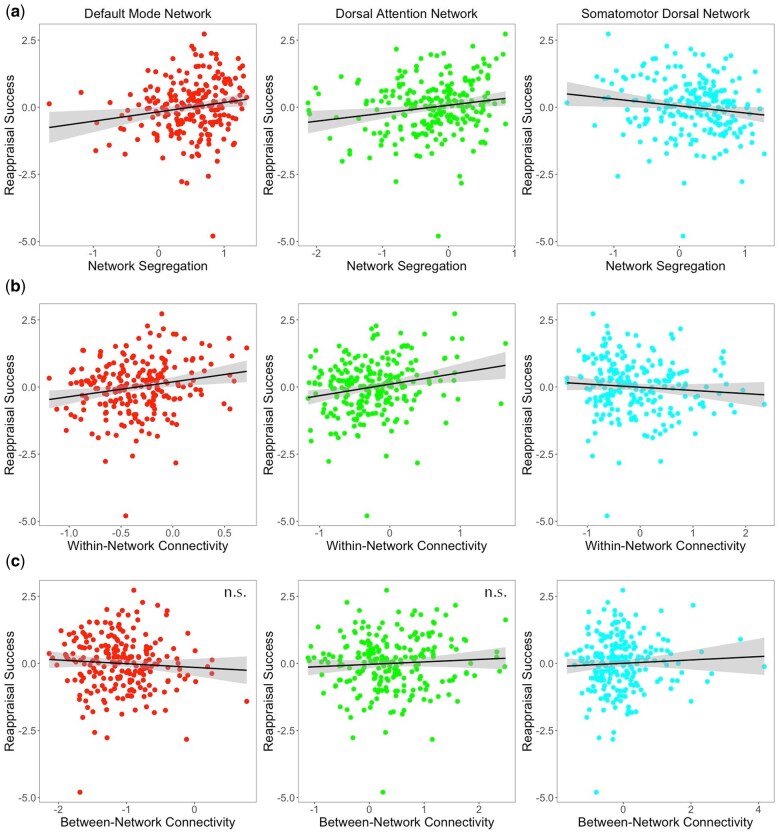
Relationship between (a) network segregation, (b) within-network connectivity, and (c) between-network connectivity (controlled for FD) with reappraisal success (controlled for age) across the three brain networks. The DMN and DAN showed a positive effect, with greater segregation and within-network connectivity associated with better reappraisal; the between-network connectivity effect was not significant in these two networks. The SMd showed a negative effect, with greater segregation and within-network connectivity associated with worse reappraisal; greater between-network connectivity of the SMd was associated with better reappraisal.

Separate linear models were fit predicting reappraisal success from network segregation (controlled for FD) of all 13 networks, with linear and quadratic effects of age included as covariates. All models yielded a significant negative quadratic effect of age on reappraisal success (Supplemental Table S2). Yet only for three networks was there a significant effect of segregation: the DMN (*F*(3, 223) = 5.725, *P* < .001, *R*^2^ = .072), DAN (*F*(3, 223) = 6.32, *P* < .001, *R*^2^ = .078), and SMd (*F*(3, 223) = 5.02, *P* = .002, *R*^2^ = .063). In the DMN and DAN models, there was a positive effect of segregation, indicating that greater segregation was related to better reappraisal success (i.e. a larger reduction in negative ratings). On the other hand, in the SMd model there was a negative effect of segregation, indicating that greater segregation was related to worse reappraisal success (Table 1 and Fig. 2).

**Table 1.** Models predicting reappraisal success from segregation and age for networks with significant effects of segregation.

Network	*B*	SE	*t*-value	*P*-value
DMN (*F*(3, 223) = 5.725, *P* = .006, *R* ^2^ = .072)				
Intercept	0.08	0.12	0.66	.509
**Segregation**	**0.35**	**0.13**	**2.67**	.008**
Age (linear)	−0.02	0.07	−0.23	.819
**Age (quadratic)**	−**0.25**	**0.09**	−**2.98**	.003**
DAN (*F*(3, 223) = 6.32, *P* = .006, *R* ^2^ = .078)				
**Intercept**	**0.32**	**0.11**	**2.87**	.004**
**Segregation**	**0.34**	**0.11**	**2.97**	.003**
Age (linear)	0.03	0.07	0.40	.686
**Age (quadratic)**	−**0.24**	**0.08**	−**2.79**	.006**
Somatomotor Dorsal (*F*(3, 223) = 5.02, *P* = .009, *R* ^2^ = .063)				
**Intercept**	**0.29**	**0.11**	**2.60**	.010**
**Segregation**	−**0.26**	**0.11**	−**2.26**	.025*
Age (linear)	−0.04	0.07	−0.61	.545
**Age (quadratic)**	−**0.24**	**0.09**	−**2.83**	.005**

Overall model *P*-values are FDR-corrected across the 13 networks. Bold font indicates a significant effect. SE = standard error.

** *P* < .01,

* *P* < .05.

Next, in each of these three networks, linear models were fit predicting reappraisal success from within-network and between-network connectivity separately, as well as age and the interactions between age and connectivity, to determine which factor was driving the segregation effects. In the DMN (*F*(6, 220) = 3.47, *P* = .003, *R^2^* = .087) and DAN (*F*(6, 220) = 4.28, *P* < .001, *R^2^* = .105), within-network connectivity was a significant predictor of reappraisal success (Table 2 and Fig. 2), such that greater within-network connectivity was associated with better reappraisal success. Between-network connectivity was not significant for either of these networks, nor were the interactions with age.

**Table 2.** Models predicting reappraisal success from between- and within-network connectivity.

Network	*B*	SE	*t*-value	*P*-value
DMN (*F*(6, 220) = 3.47, *P* = .003, *R* ^2^ = .087)				
Intercept	0.29	0.18	1.59	.113
Between-network	−0.14	0.14	−0.99	.324
**Within-network**	**0.57**	**0.19**	**3.05**	**.003** **
Age (linear)	0.03	0.18	0.17	.863
**Age (quadratic)**	−**0.24**	**0.09**	−**2.64**	.009**
Between*Age	0.04	0.14	0.33	.743
Within*Age	−0.06	0.19	−0.35	.730
DAN (*F*(6, 220) = 4.28, *P* < .001, *R^2^* = .105)				
**Intercept**	**0.36**	**0.13**	**2.69**	.008**
Between-network	−0.08	0.11	−0.75	.455
**Within-network**	**0.53**	**0.16**	**3.28**	.001**
Age (linear)	−0.05	0.11	−0.49	.622
**Age (quadratic)**	−**0.24**	**0.09**	−**2.71**	.007**
Between*Age	0.04	0.11	0.38	.707
Within*Age	−0.26	0.20	−1.33	.185
Somatomotor dorsal (*F*(6, 220) = 2.78, *P* = .013, *R* ^2^ = .070)				
Intercept	0.20	0.11	1.87	.062^+^
**Between-network**	**0.25**	**0.12**	**2.13**	.034*
**Within-network**	−**0.31**	**0.13**	−**2.37**	.019*
Age (linear)	−0.05	0.08	−0.61	.542
**Age (quadratic)**	−**0.21**	**0.09**	−**2.32**	.021*
Between*Age	0.02	0.12	0.19	.850
Within*Age	−0.03	0.12	−0.25	.804

Bold font indicates a significant effect. DAN = dorsal attention network; DMN = default mode network; SE = standard error.

** *P* < .01,

* *P* < .05,

+ *P* < .10.

**Figure 3.**
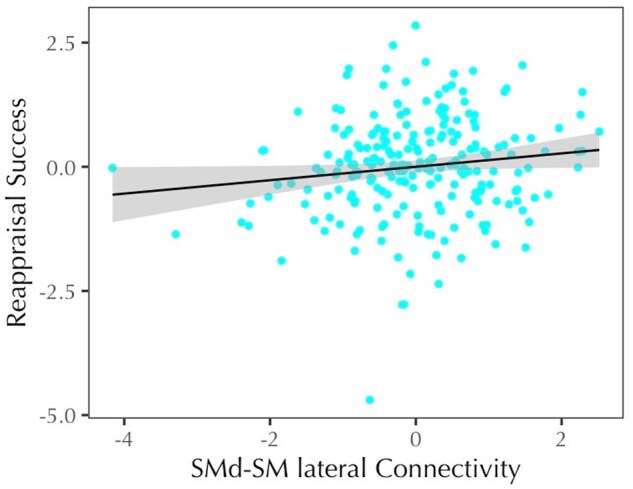
In the selected model including a quadratic effect of age and within-SMd connectivity (Table 3), stronger between-network connectivity of the SMd and SM lateral networks predicted better reappraisal.

In the SMd model (*F*(6, 220) = 2.78, *P* = .013, *R^2^* = .070), within-network connectivity showed a significant negative effect, such that lower within-network connectivity was associated with better reappraisal success. Additionally, between-network connectivity showed a significant positive effect (Table 2 and Fig. 2). The between-network effect for the SMd was probed further to investigate which network connections were involved. A linear model was fit predicting reappraisal success from within-network connectivity of the SMd, connectivity between SMd and each of the other 12 networks, and age. The initial model was then reduced by removing non-significant predictors to minimize the model AIC. The final model (*F*(3, 223) = 5.23, *P* = .002, *R^2^* = .066) included the quadratic effect of age, within-network connectivity, and connectivity between SMd and lateral SM; greater connectivity between SMd and lateral SM predicted better reappraisal success (Fig. 3). Bootstrap resampling of variable selection indicated that each of these predictors were included in at least 75% (77%–100%) of models, while other predictors were included in less than 53% of models. Bootstrapped median estimates of the selected predictors’ model coefficients were similar to the full model estimates, suggesting limited bias in model selection (Table 3).

**Table 3.** Details of the full and selected models predicting reappraisal success from connectivity of the SMd.

	Full model			Selected model						
Predictors	Estimate	SE	Bootstrap inclusion frequency (%)	Estimate	SE	RMSD ratio	Relative conditional bias	Bootstrap median	Bootstrap 2.5th percentile	Bootstrap 97.5th percentile
**Intercept**	**0.76**	**0.43**	**100**	**0.55**	**0.18**	**0.97**	−**3.89**	**0.71**	−**0.06**	**1.50**
**Within**	−**0.27**	**0.14**	**90.9**	−**0.22**	**0.09**	**0.98**	−**0.97**	−**0.25**	−**0.5**	**0.00**
**Age (quadratic)**	−**0.19**	**0.09**	**82.4**	−**0.26**	**0.08**	**1.15**	**23.19**	−**0.2**	−**0.38**	**0.00**
**Between SMd-SMl**	**0.22**	**0.15**	**77.0**	**0.27**	**0.13**	**1.03**	**28.85**	**0.23**	**0.00**	**0.50**
Between SMd-DMN	0.49	0.42	52.9	0	0	1.07	64.47	0.48	0.00	1.30
Between SMd-CO	0.28	0.29	51.3	0	0	0.98	50.71	0	−0.23	0.81
Between SMd-AUD	0.04	0.13	50.3	0	0	0.87	66.33	0	−0.21	0.26
Between SMd-VIS	0.18	0.19	39.6	0	0	1.02	79.56	0	0.00	0.52
Age (linear)	−0.06	0.08	37.4	0	0	1.09	128.18	0	−0.23	0.00
Between SMd-SAL	0.00	0.37	36.9	0	0	0.85	610.70	0	−0.68	0.73
Between SMd-DAN	−0.07	0.23	27.0	0	0	0.79	216.64	0	−0.48	0.29
Between SMd-FPN	−0.26	0.55	25.7	0	0	0.90	116.17	0	−1.32	0.81
Between SMd-Reward	0.06	0.30	25.5	0	0	0.80	226.5	0	−0.52	0.62
Between SMd-MTL	0.07	0.17	23.7	0	0	0.77	151.11	0	−0.21	0.36
Between SMd-VAN	−0.03	0.16	21.3	0	0	0.79	−43.43	0	−0.31	0.30
Between SMd-PM	0.02	0.33	18.0	0	0	0.74	−193.97	0	−0.63	0.60

Bold font indicates predictors included in the selected model. AUD = auditory; CO = cingulo-opercular; DAN = dorsal attention network; DMN = default mode network; FPN = fronto-parietal network; MTL = medial temporal lobe; PM = parietal medial; SAL = salience; SMd = somatomotor-dorsal; SMl = somatomotor-lateral; VAN = ventral attention network; VIS = visual; SE = standard error; RMSD = root mean squared difference (variance).

### Comparison with task-based activation

**Figure 4.**
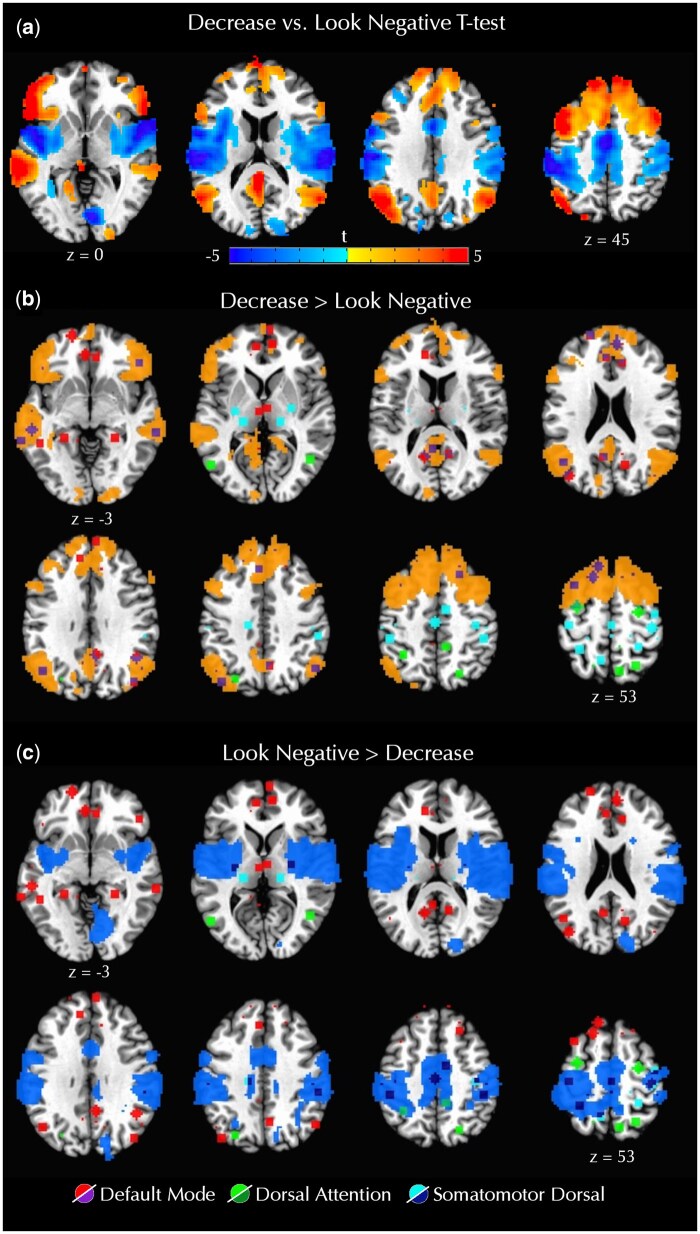
Brain map of the Decrease—Look Negative *t*-test for the fMRI task [(a) voxel-wise *P* < .005, alpha < .05]. In the lower panels, ROIs from the three networks identified in the resting-state analysis (DMN, DAN, and SMd) are overlaid on a binarized version of the map in panel (a), with clusters showing greater activation for Decrease trials (b) or greater activation for Look Negative trials (c). This visual comparison suggests that the DMN showed the most overlap with the reappraisal task activation during Decrease trials, while the SMd showed the most overlap with the task activation during Look Negative trials. The DAN showed minimal overlap with both activation maps, suggesting no reappraisal task-related activation in this network.

**Figure 5.**
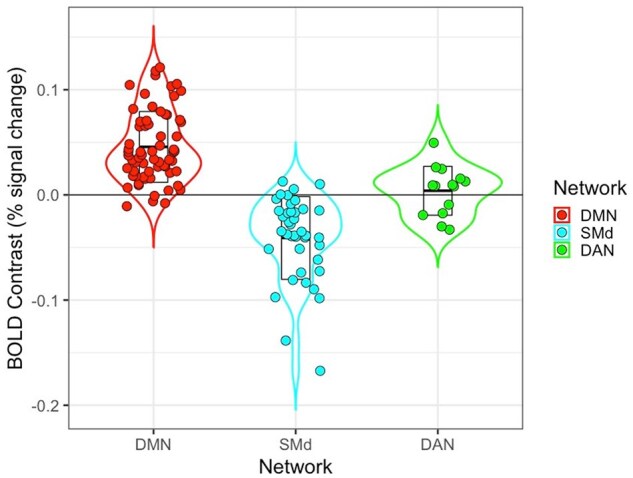
Task BOLD values for the Decrease—Look Negative contrast in each ROI across the three resting-state networks. The DMN ROIs mostly showed greater activation for Decrease trials, the SMd ROIs showed greater activation for Look Negative trials, and the DAN ROIs showed little to no difference between conditions.

A one sample *t*-test identified bilateral regions of prefrontal, parietal, and temporal cortex that were more active during regulation (Decrease trials) than viewing of negative images (Fig. 4a and Table S3), consistent with prior work. For the natural viewing condition (Look Negative trials), there was greater activation in bilateral precentral and postcentral gyri extending to the posterior insula. These group activation maps were then spatially overlaid with the ROIs comprising the three networks identified in the resting-state analysis. This visual comparison illustrated which of these regions were also involved during the reappraisal task. For the clusters that were more activated during Decrease trials (i.e. support reappraisal), the greatest overlap was observed with ROIs from the DMN, with minor overlap for the DAN and no overlap for the SMd (Fig. 4b). For the clusters that were more activated during Look Negative trials (i.e. support emotional reactivity), the greatest overlap was observed with ROIs from the SMd, with minor overlap for the DAN and no overlap for the DMN (Fig. 4c). To quantify the apparent overlap between the resting-state network ROIs and reappraisal task-based activation, the unthresholded beta values were extracted for the Decrease vs. Look Negative contrast from each participant and averaged for the ROIs in each network. These results confirmed the pattern observed in the overlap maps. Specifically, most DMN ROIs exhibited stronger activation for Decrease compared to Look Negative trials (*t*(64) = 10.93, *P* < .001), while most SMd ROIs exhibited stronger activation for Look Negative trials (*t*(39) = −6.59, *P* < .001); DAN ROIs showed minimal activation differences between task conditions (*t*(13) = 0.64, *P* = .536; Fig. 5).

## Discussion

In this study, we investigated how cognitive reappraisal success across the lifespan related to the functional organization of 13 brain networks measured during resting-state fMRI. Behaviourally, reappraisal success followed an inverted u-shape curve with respect to age, with young adults reporting the greatest reduction in negative feelings during emotion regulation. In terms of RSFC, three brain networks exhibited a relationship with reappraisal success: DMN and DAN showed a positive effect, with greater segregation associated with better reappraisal success, whereas SMd showed the reverse pattern. In all three networks, these segregation effects were driven by differences in within-network connectivity, and in the SMd, between-network connectivity with the lateral SM network also contributed. Additionally, the significant resting-state networks partially overlapped with regions showing task-based (de)activation during reappraisal, suggesting that the functional organization of both reappraisal-specific and general attention regions support emotion regulation ability.

### Default mode network in emotion (re)appraisal

In the current analysis of RSFC, greater network segregation of the DMN was associated with better reappraisal success. Individuals with a more segregated DMN, driven by stronger within-DMN connectivity, were able to reduce their negative feelings during the reappraisal task to a greater degree than those with a less segregated DMN. Prior work identified the DMN as relevant for emotion processing, including cognitive reappraisal specifically (Bo et al. 2024) and thinking about social/affective states generally (Helion et al. 2019, Silvers and Moreira 2019). Many of the ROIs comprising the DMN were also activated during the reappraisal task itself, including those in superior PFC, ventrolateral PFC, middle temporal gyrus, and lateral parietal cortex. A number of these regions correspond to those previously identified in studies of reappraisal, though not necessarily as part of the DMN (Buhle et al. 2014, Frank et al. 2014, Morawetz et al. 2024). This activation indicates that not only resting-state organization, but also task recruitment of the network supports reappraisal.

The DMN, therefore, may contribute to the formation of new appraisals of emotional images, perhaps through mentalizing about one’s own emotional state or that of people depicted in the scenes, or by reflecting about autobiographical experiences that could inform a given situation (Raichle 2015, Helion et al. 2019, Menon 2023). Stronger internal connectivity with greater segregation from other networks may indicate a more cohesive DMN that can efficiently bring forth the new appraisals with limited interference from other ongoing mental processes. The ability to generate personally relevant reappraisals via the DMN may provide a strong foundation for reshaping one’s emotional response once these reinterpretations are passed to other networks to implement additional aspects of the reappraisal process.

### Dorsal attention network supports general task engagement

Similar to the DMN, the DAN showed a positive association between network segregation during resting-state and reappraisal success, with stronger within-network connectivity supporting better reappraisal. Unlike the DMN and SMd, however, the DAN did not exhibit a clear pattern of activation for either reappraise or natural viewing trials in the emotion regulation task. While it is possible that these regions were simply not recruited by the task, it seems likely that the attentional demands of cognitive reappraisal and evaluating one’s emotional state engaged the DAN to keep attention focused across conditions. Indeed, some previous work described a role for the DAN in reappraisal (Silvers and Moreira 2019), although its proximity to regions of the fronto-parietal control network make it difficult to accurately localize previously reported activations.

Notwithstanding the lack of task-based activation in the current study, the RSFC results indicated that those participants who had a more segregated DAN with stronger within-network connectivity were better prepared to perform the reappraisal task successfully. This organization may reflect a high functional specialization of the DAN based on a history of regular engagement of attention-related regions for a variety of situations requiring an individual to direct spatial attention according to internal goals (Corbetta and Shulman 2002, Petersen and Posner 2012, Vossel et al. 2014). In the current emotion regulation task, attention and eye movements were not restricted, potentially meaning that those with stronger DAN connectivity could better direct their attention to less emotionally intense or negative aspects of the image to facilitate reappraisal. On the other hand, greater segregation from other networks may limit the DAN from being influenced as strongly by goal-irrelevant processes or the initial emotional response to the stimulus. Overall, the functional organization of the DAN seems to reflect a propensity for controlled attention that benefits reappraisal without this network being specifically engaged during reappraisal trials.

### Somatomotor network and interoception

In contrast to the two networks described above, the SMd exhibited a negative relationship between network segregation and reappraisal success. Greater segregation was predictive of worse reappraisal, which was driven by greater within-network connectivity and weaker connectivity between the SMd and lateral SM network. Most of the ROIs in the SMd showed greater task activation during natural viewing compared to reappraisal, including the SMA, precentral gyrus, postcentral gyrus, and cerebellum. This pattern of results is consistent with previous literature, which has demonstrated decreased task activation in postcentral gyrus and insula during emotion downregulation (Min et al. 2022). Prior work has also shown that somatosensory cortex is modifiable according to emotion regulation goals, with greater activation correlated with worse reappraisal performance (Bo et al. 2024).

Sensorimotor regions are associated with interoceptive functions that may support the physiological response or motor preparation aspects of an emotional experience (Adolfi et al. 2017, Min et al. 2022). Moreover, bodily awareness may be an important factor in assessing one’s own emotional state in order to regulate it (Ahmadi Ghomroudi et al. 2024), resulting in reduced activation during reappraisal. Stronger within-SMd connectivity may impair successful reappraisal if the interoceptive signals are being reinforced and not modulated sufficiently by control networks. One previous study assessing resting-state and reappraisal using a data-driven approach (Ahmadi Ghomroudi et al. 2024) also reported that a sensorimotor network was related to reappraisal *tendency*. Together with the current findings, this suggests that focusing only on specific task-based ROIs or connections that were emphasized by prior literature may overlook the role of distributed coordination among widespread brain regions.

### Cognitive reappraisal across the lifespan

Behaviourally, the current results demonstrated that young adults were most successful at using reappraisal to reduce negative feelings during emotion regulation. These differences were driven by ratings during the Decrease trials, yet there were no age-related differences in Look Negative trial ratings, despite prior reports of a dampening of negative reactivity in ageing (Mather 2016). Furthermore, there were not any interactions with age in how RSFC related to reappraisal success. This lack of an effect may be due to the wide age range in the sample, resulting in relatively few participants for any given age and low statistical power to detect weaker interactions. It does suggest, however, that individuals with the highest reappraisal ability relied upon broadly similar neural mechanisms to perform the necessary emotion regulatory processing, regardless of age.

### Limitations

Although the current reappraisal task design followed a standard approach to measuring emotion regulation ability, it is important to acknowledge that there are limitations to this paradigm. Cognitive reappraisal of static images within a laboratory setting may rely upon neural mechanisms that differ from those that come online in real-world contexts (Wilson-Mendenhall and Holmes 2023). Reappraisal ability can also differ from reappraisal tendency (Silvers and Moreira 2019) and there may be unique age-related RSFC differences relative to how emotion regulation strategies are selected and implemented across the lifespan. Additionally, the resting-state scans in our study were collected primarily after completion of the emotion regulation task (for children, the resting-state was split into multiple shorter scans both before and after the task). It is known that prior task context can influence resting-state activity, although broad patterns of network topology tend to be preserved (Pyka et al. 2009, Grigg and Grady 2010, Tailby et al. 2015). Accordingly, the observed differences in RSFC with respect to reappraisal success may partially reflect dynamic differences in post-task processing rather than stable intrinsic brain organization alone. Nonetheless, such differences are still informative about the networks that are relevant to reappraisal ability, and future research should investigate how sensitive this relationship is to task context.

Another potential limitation to consider is that we utilized global signal regression during preprocessing, which improves resting-state signal fidelity but also introduces artefactual negative correlations (Murphy and Fox 2017). Therefore, as in prior work (Chan et al. 2014, Zhang et al. 2023b, Pierce et al. 2024), the network segregation analysis examined only positive correlations between ROIs, meaning that anti-correlated between-network connectivity cannot be considered here. Given that the network segregation measure aggregates connectivity across nodes and networks and relies on a subtraction of between and within network connectivity, the inclusion of negative correlations could lead to mischaracterization of true segregation. Thus, although there may be important information in well-documented anti-correlations (e.g. PFC and amygdala) that are excluded here, targeted analyses of these specific regions are better suited to characterizing their relationship with behaviour than this whole-brain, cross-network segregation approach.

## Conclusion

In this study, we found that task-based reappraisal success in individuals from 6 to 80 years old was related to intrinsic organization in three brain networks: the DMN, DAN, and SMd. Additionally, the DMN generally showed greater activation during reappraisal trials, the SMd showed greater activation during natural viewing trials, and the DAN showed no difference between conditions. Collectively, the present findings demonstrated that RSFC is critical for reappraisal success, even in regions that may not be specifically recruited during task performance. A better understanding of how brain organization facilitates emotion regulation has implications for a wide range of studies covering social-affective topics such as stress responses, personality traits, and mood disorders characterized by emotion dysregulation.

## Supplementary Material

nsaf055_Supplementary_Data

## Data Availability

Raw data for participants up to the age of 35 years are available on the NIH Data Archive (https://nda.nih.gov/). Correlation matrices from all participants are publicly available on OSF (https://osf.io/xv7yz/). Network segregation scripts are available at https://gitlab.com/wiglab/system-segregation-and-graph-tools.
