# Supplementary material for: Segregation of three resting-state brain networks predicts reappraisal success across the lifespan
Source: Soc Cogn Affect Neurosci. 2025 May 21;20(1):nsaf055. doi: 10.1093/scan/nsaf055 (PMC13223757; doi:10.1093/scan/nsaf055)
Supplement: nsaf055_Supplementary_Data [file nsaf055_supplementary_data.docx]

SEGREGATION OF THREE RESTING-STATE BRAIN NETWORKS PREDICTS REAPPRAISAL SUCCESS ACROSS THE LIFESPAN

Supplemental Material

Jordan E. Pierce & Maital Neta

**Table S1**. List of ROI coordinates (MNI) and associated network labels from Seitzman et al. (2020).

| **Network Number** | **Network Name** | **x** | **y** | **z** |
| --- | --- | --- | --- | --- |
| 1 | Unassigned | -56 | -45 | -24 |
| 1 | Unassigned | -25 | -98 | -12 |
| 1 | Unassigned | 8 | 41 | -24 |
| 1 | Unassigned | 27 | -97 | -13 |
| 1 | Unassigned | 49 | -3 | -38 |
| 1 | Unassigned | 52 | -34 | -27 |
| 1 | Unassigned | 55 | -31 | -17 |
| 1 | Unassigned | -58 | -26 | -15 |
| 1 | Unassigned | 65 | -24 | -19 |
| 1 | Unassigned | -50 | -7 | -39 |
| 1 | Unassigned | 34 | 38 | -12 |
| 1 | Unassigned | -47 | -51 | -21 |
| 2 | Somatomotor Dorsal | -7 | -52 | 61 |
| 2 | Somatomotor Dorsal | 0 | -15 | 47 |
| 2 | Somatomotor Dorsal | -54 | -23 | 43 |
| 2 | Somatomotor Dorsal | -45 | -32 | 47 |
| 2 | Somatomotor Dorsal | -40 | -19 | 54 |
| 2 | Somatomotor Dorsal | -38 | -27 | 69 |
| 2 | Somatomotor Dorsal | -38 | -15 | 69 |
| 2 | Somatomotor Dorsal | -29 | -43 | 61 |
| 2 | Somatomotor Dorsal | -23 | -30 | 72 |
| 2 | Somatomotor Dorsal | -21 | -31 | 61 |
| 2 | Somatomotor Dorsal | -16 | -46 | 73 |
| 2 | Somatomotor Dorsal | -14 | -18 | 40 |
| 2 | Somatomotor Dorsal | -13 | -17 | 75 |
| 2 | Somatomotor Dorsal | -7 | -21 | 65 |
| 2 | Somatomotor Dorsal | -7 | -33 | 72 |
| 2 | Somatomotor Dorsal | 2 | -28 | 60 |
| 2 | Somatomotor Dorsal | 3 | -17 | 58 |
| 2 | Somatomotor Dorsal | 10 | -2 | 45 |
| 2 | Somatomotor Dorsal | 10 | -46 | 73 |
| 2 | Somatomotor Dorsal | 10 | -17 | 74 |
| 2 | Somatomotor Dorsal | 13 | -33 | 75 |
| 2 | Somatomotor Dorsal | 20 | -29 | 60 |
| 2 | Somatomotor Dorsal | 22 | -42 | 69 |
| 2 | Somatomotor Dorsal | 29 | -17 | 71 |
| 2 | Somatomotor Dorsal | 29 | -39 | 59 |
| 2 | Somatomotor Dorsal | 38 | -17 | 45 |
| 2 | Somatomotor Dorsal | 42 | -20 | 55 |
| 2 | Somatomotor Dorsal | 44 | -8 | 57 |
| 2 | Somatomotor Dorsal | 47 | -30 | 49 |
| 2 | Somatomotor Dorsal | 50 | -20 | 42 |
| 2 | Somatomotor Dorsal | 19 | -8 | 64 |
| 2 | Somatomotor Dorsal | 54 | -28 | 34 |
| 2 | Somatomotor Dorsal | 29 | -8 | 8 |
| 2 | Somatomotor Dorsal | -28 | -10 | 9 |
| 2 | Somatomotor Dorsal | 16 | -22 | 9 |
| 2 | Somatomotor Dorsal | -19 | -23 | 10 |
| 2 | Somatomotor Dorsal | -6 | -74 | -42 |
| 2 | Somatomotor Dorsal | 8 | -72 | -39 |
| 2 | Somatomotor Dorsal | -12 | -44 | -18 |
| 2 | Somatomotor Dorsal | 12 | -44 | -18 |
| 3 | Somatomotor Lateral | -53 | -10 | 24 |
| 3 | Somatomotor Lateral | -49 | -11 | 35 |
| 3 | Somatomotor Lateral | 36 | -9 | 14 |
| 3 | Somatomotor Lateral | 51 | -6 | 32 |
| 3 | Somatomotor Lateral | 66 | -8 | 25 |
| 3 | Somatomotor Lateral | 28 | -7 | -5 |
| 3 | Somatomotor Lateral | -28 | -10 | -4 |
| 3 | Somatomotor Lateral | 14 | -19 | 0 |
| 3 | Somatomotor Lateral | -14 | -20 | 1 |
| 3 | Somatomotor Lateral | -10 | -62 | -18 |
| 3 | Somatomotor Lateral | 10 | -62 | -18 |
| 4 | Cingulo-Opercular | -51 | 8 | -2 |
| 4 | Cingulo-Opercular | -45 | 0 | 9 |
| 4 | Cingulo-Opercular | -34 | 3 | 4 |
| 4 | Cingulo-Opercular | -16 | -5 | 71 |
| 4 | Cingulo-Opercular | -10 | -2 | 42 |
| 4 | Cingulo-Opercular | -5 | 18 | 34 |
| 4 | Cingulo-Opercular | -3 | 2 | 53 |
| 4 | Cingulo-Opercular | 7 | 8 | 51 |
| 4 | Cingulo-Opercular | 13 | -1 | 70 |
| 4 | Cingulo-Opercular | 36 | 10 | 1 |
| 4 | Cingulo-Opercular | 37 | 1 | -4 |
| 4 | Cingulo-Opercular | 49 | 8 | -1 |
| 4 | Cingulo-Opercular | -30 | -27 | 12 |
| 4 | Cingulo-Opercular | 42 | 0 | 47 |
| 4 | Cingulo-Opercular | 36 | 22 | 3 |
| 4 | Cingulo-Opercular | -39 | 51 | 17 |
| 4 | Cingulo-Opercular | 31 | 33 | 26 |
| 4 | Cingulo-Opercular | 31 | 56 | 14 |
| 4 | Cingulo-Opercular | 37 | 32 | -2 |
| 4 | Cingulo-Opercular | 25 | 5 | 7 |
| 4 | Cingulo-Opercular | -25 | 8 | 8 |
| 4 | Cingulo-Opercular | 19 | -5 | -4 |
| 4 | Cingulo-Opercular | -19 | -5 | -3 |
| 4 | Cingulo-Opercular | 13 | -14 | 12 |
| 4 | Cingulo-Opercular | -15 | -14 | 12 |
| 4 | Cingulo-Opercular | 10 | -8 | 2 |
| 4 | Cingulo-Opercular | -9 | -10 | 0 |
| 4 | Cingulo-Opercular | 32 | -49 | -51 |
| 4 | Cingulo-Opercular | -34 | -42 | -44 |
| 4 | Cingulo-Opercular | -33 | -51 | -50 |
| 5 | Auditory | -53 | -22 | 23 |
| 5 | Auditory | 43 | -23 | 20 |
| 5 | Auditory | 59 | -17 | 29 |
| 5 | Auditory | -60 | -25 | 14 |
| 5 | Auditory | -55 | -9 | 12 |
| 5 | Auditory | -50 | -34 | 26 |
| 5 | Auditory | -49 | -26 | 5 |
| 5 | Auditory | -38 | -33 | 17 |
| 5 | Auditory | 32 | -26 | 13 |
| 5 | Auditory | 56 | -5 | 13 |
| 5 | Auditory | 58 | -16 | 7 |
| 5 | Auditory | 65 | -33 | 20 |
| 6 | Default Mode | -68 | -23 | -16 |
| 6 | Default Mode | -68 | -41 | -5 |
| 6 | Default Mode | -58 | -30 | -4 |
| 6 | Default Mode | -56 | -13 | -10 |
| 6 | Default Mode | -53 | 3 | -27 |
| 6 | Default Mode | -49 | -42 | 1 |
| 6 | Default Mode | -46 | -61 | 21 |
| 6 | Default Mode | -46 | 31 | -13 |
| 6 | Default Mode | -44 | -65 | 35 |
| 6 | Default Mode | -44 | 12 | -34 |
| 6 | Default Mode | -41 | -75 | 26 |
| 6 | Default Mode | -39 | -75 | 44 |
| 6 | Default Mode | -35 | 20 | 51 |
| 6 | Default Mode | -20 | 45 | 39 |
| 6 | Default Mode | -20 | 64 | 19 |
| 6 | Default Mode | -18 | 63 | -9 |
| 6 | Default Mode | -16 | 29 | 53 |
| 6 | Default Mode | -13 | -40 | 1 |
| 6 | Default Mode | -11 | -56 | 16 |
| 6 | Default Mode | -10 | 39 | 52 |
| 6 | Default Mode | -10 | 55 | 39 |
| 6 | Default Mode | -11 | 45 | 8 |
| 6 | Default Mode | -8 | 48 | 23 |
| 6 | Default Mode | -7 | -55 | 27 |
| 6 | Default Mode | -7 | 51 | -1 |
| 6 | Default Mode | -3 | -49 | 13 |
| 6 | Default Mode | -2 | -37 | 44 |
| 6 | Default Mode | -3 | 44 | -9 |
| 6 | Default Mode | -2 | 38 | 36 |
| 6 | Default Mode | -3 | 42 | 16 |
| 6 | Default Mode | 6 | 67 | -4 |
| 6 | Default Mode | 6 | -59 | 35 |
| 6 | Default Mode | 6 | 54 | 16 |
| 6 | Default Mode | 6 | 64 | 22 |
| 6 | Default Mode | 8 | -48 | 31 |
| 6 | Default Mode | 8 | 42 | -5 |
| 6 | Default Mode | 8 | 48 | -15 |
| 6 | Default Mode | 9 | 54 | 3 |
| 6 | Default Mode | 11 | -54 | 17 |
| 6 | Default Mode | 13 | 55 | 38 |
| 6 | Default Mode | 13 | 30 | 59 |
| 6 | Default Mode | 12 | 36 | 20 |
| 6 | Default Mode | 15 | -63 | 26 |
| 6 | Default Mode | 22 | 39 | 39 |
| 6 | Default Mode | 23 | 33 | 48 |
| 6 | Default Mode | 43 | -72 | 28 |
| 6 | Default Mode | 46 | 16 | -30 |
| 6 | Default Mode | 47 | -50 | 29 |
| 6 | Default Mode | 49 | 35 | -12 |
| 6 | Default Mode | 52 | -59 | 36 |
| 6 | Default Mode | 52 | -2 | -16 |
| 6 | Default Mode | 52 | 7 | -30 |
| 6 | Default Mode | 65 | -12 | -19 |
| 6 | Default Mode | 65 | -31 | -9 |
| 6 | Default Mode | -34 | -38 | -16 |
| 6 | Default Mode | -25 | -39 | -2 |
| 6 | Default Mode | 25 | -37 | -2 |
| 6 | Default Mode | 3 | -8 | 8 |
| 6 | Default Mode | -3 | -10 | 9 |
| 6 | Default Mode | -32 | -78 | -38 |
| 6 | Default Mode | 32 | -81 | -38 |
| 6 | Default Mode | -24 | -76 | -28 |
| 6 | Default Mode | 24 | -76 | -28 |
| 6 | Default Mode | -6 | -51 | -41 |
| 6 | Default Mode | 8 | -50 | -40 |
| 7 | Parietal Medial | 4 | -48 | 51 |
| 7 | Parietal Medial | -7 | -71 | 42 |
| 7 | Parietal Medial | -2 | -35 | 31 |
| 7 | Parietal Medial | 2 | -24 | 30 |
| 7 | Parietal Medial | 11 | -66 | 42 |
| 8 | Visual | -12 | -95 | -13 |
| 8 | Visual | 8 | -91 | -7 |
| 8 | Visual | 17 | -91 | -14 |
| 8 | Visual | -47 | -76 | -10 |
| 8 | Visual | -42 | -74 | 0 |
| 8 | Visual | -40 | -88 | -6 |
| 8 | Visual | -33 | -79 | -13 |
| 8 | Visual | -28 | -79 | 19 |
| 8 | Visual | -26 | -90 | 3 |
| 8 | Visual | -24 | -91 | 19 |
| 8 | Visual | -18 | -68 | 5 |
| 8 | Visual | -16 | -77 | 34 |
| 8 | Visual | -16 | -52 | -1 |
| 8 | Visual | -15 | -72 | -8 |
| 8 | Visual | -14 | -91 | 31 |
| 8 | Visual | -8 | -81 | 7 |
| 8 | Visual | -3 | -81 | 21 |
| 8 | Visual | 6 | -72 | 24 |
| 8 | Visual | 6 | -81 | 6 |
| 8 | Visual | 8 | -72 | 11 |
| 8 | Visual | 15 | -87 | 37 |
| 8 | Visual | 15 | -77 | 31 |
| 8 | Visual | 18 | -47 | -10 |
| 8 | Visual | 20 | -66 | 2 |
| 8 | Visual | 20 | -86 | -2 |
| 8 | Visual | 24 | -87 | 24 |
| 8 | Visual | 26 | -79 | -16 |
| 8 | Visual | 27 | -59 | -9 |
| 8 | Visual | 29 | -77 | 25 |
| 8 | Visual | 37 | -84 | 13 |
| 8 | Visual | 37 | -81 | 1 |
| 8 | Visual | 40 | -72 | 14 |
| 8 | Visual | 42 | -66 | -8 |
| 8 | Visual | 43 | -78 | -12 |
| 8 | Visual | 19 | -29 | 1 |
| 8 | Visual | -19 | -29 | 2 |
| 8 | Visual | 0 | -74 | -25 |
| 9 | Fronto-Parietal | -53 | -49 | 43 |
| 9 | Fronto-Parietal | -47 | 11 | 23 |
| 9 | Fronto-Parietal | -44 | 2 | 46 |
| 9 | Fronto-Parietal | -42 | 38 | 21 |
| 9 | Fronto-Parietal | -42 | -55 | 45 |
| 9 | Fronto-Parietal | -42 | 25 | 30 |
| 9 | Fronto-Parietal | -41 | 6 | 33 |
| 9 | Fronto-Parietal | -42 | 45 | -2 |
| 9 | Fronto-Parietal | -34 | 55 | 4 |
| 9 | Fronto-Parietal | -28 | -58 | 48 |
| 9 | Fronto-Parietal | -23 | 11 | 64 |
| 9 | Fronto-Parietal | -3 | 26 | 44 |
| 9 | Fronto-Parietal | 24 | 45 | -15 |
| 9 | Fronto-Parietal | 32 | 14 | 56 |
| 9 | Fronto-Parietal | 33 | -53 | 44 |
| 9 | Fronto-Parietal | 34 | 54 | -13 |
| 9 | Fronto-Parietal | 37 | -65 | 40 |
| 9 | Fronto-Parietal | 38 | 43 | 15 |
| 9 | Fronto-Parietal | 40 | 18 | 40 |
| 9 | Fronto-Parietal | 44 | -53 | 47 |
| 9 | Fronto-Parietal | 43 | 49 | -2 |
| 9 | Fronto-Parietal | 47 | 10 | 33 |
| 9 | Fronto-Parietal | 48 | 25 | 27 |
| 9 | Fronto-Parietal | 49 | -42 | 45 |
| 9 | Fronto-Parietal | 58 | -53 | -14 |
| 9 | Fronto-Parietal | 48 | 22 | 10 |
| 9 | Fronto-Parietal | 55 | -45 | 37 |
| 9 | Fronto-Parietal | 14 | -1 | 18 |
| 9 | Fronto-Parietal | -15 | -2 | 19 |
| 9 | Fronto-Parietal | -10 | -78 | -28 |
| 9 | Fronto-Parietal | 10 | -78 | -28 |
| 9 | Fronto-Parietal | -34 | -72 | -48 |
| 9 | Fronto-Parietal | 34 | -72 | -48 |
| 9 | Fronto-Parietal | -31 | -66 | -30 |
| 9 | Fronto-Parietal | 32 | -63 | -30 |
| 9 | Fronto-Parietal | 40 | -44 | -38 |
| 10 | Salience | -35 | 20 | 0 |
| 10 | Salience | -28 | 52 | 21 |
| 10 | Salience | -11 | 26 | 25 |
| 10 | Salience | -1 | 15 | 44 |
| 10 | Salience | 0 | 30 | 27 |
| 10 | Salience | 5 | 23 | 37 |
| 10 | Salience | 10 | 22 | 27 |
| 10 | Salience | 26 | 50 | 27 |
| 10 | Salience | 34 | 16 | -8 |
| 10 | Salience | 12 | 18 | 7 |
| 10 | Salience | -13 | 17 | 7 |
| 10 | Salience | 44 | -60 | -30 |
| 10 | Salience | -44 | -60 | -30 |
| 11 | Ventral Attention | 51 | -29 | -4 |
| 11 | Ventral Attention | -56 | -50 | 10 |
| 11 | Ventral Attention | -55 | -40 | 14 |
| 11 | Ventral Attention | 52 | -33 | 8 |
| 11 | Ventral Attention | 56 | -46 | 11 |
| 11 | Ventral Attention | -49 | 25 | -1 |
| 11 | Ventral Attention | -10 | 11 | 67 |
| 11 | Ventral Attention | 53 | 33 | 1 |
| 11 | Ventral Attention | 54 | -43 | 22 |
| 11 | Ventral Attention | 25 | 2 | -1 |
| 11 | Ventral Attention | -28 | -1 | -3 |
| 12 | Dorsal Attention | -27 | -71 | 37 |
| 12 | Dorsal Attention | -37 | -29 | -26 |
| 12 | Dorsal Attention | 46 | -47 | -17 |
| 12 | Dorsal Attention | 11 | -39 | 50 |
| 12 | Dorsal Attention | -52 | -63 | 5 |
| 12 | Dorsal Attention | -42 | -60 | -9 |
| 12 | Dorsal Attention | -33 | -46 | 47 |
| 12 | Dorsal Attention | -32 | -1 | 54 |
| 12 | Dorsal Attention | -17 | -59 | 64 |
| 12 | Dorsal Attention | 10 | -62 | 61 |
| 12 | Dorsal Attention | 22 | -65 | 48 |
| 12 | Dorsal Attention | 25 | -58 | 60 |
| 12 | Dorsal Attention | 29 | -5 | 54 |
| 12 | Dorsal Attention | 46 | -59 | 4 |
| 12 | Dorsal Attention | -13 | -52 | -50 |
| 12 | Dorsal Attention | 14 | -48 | -52 |
| 13 | Medial Temporal Lobe | -31 | -10 | -36 |
| 13 | Medial Temporal Lobe | 33 | -12 | -34 |
| 13 | Medial Temporal Lobe | -26 | -12 | -22 |
| 13 | Medial Temporal Lobe | 25 | -11 | -23 |
| 14 | Reward | -31 | 19 | -19 |
| 14 | Reward | -21 | 41 | -20 |
| 14 | Reward | 24 | 32 | -18 |
| 14 | Reward | 27 | 16 | -17 |
| 14 | Reward | -20 | -2 | -22 |
| 14 | Reward | 20 | -2 | -23 |
| 14 | Reward | 13 | 17 | -5 |
| 14 | Reward | -12 | 17 | -4 |

**Table S2**. Models predicting reappraisal success from network segregation and age for networks with no effect of segregation.

| **Network** | ***B*** | **SE** | ***t*-value** | ***p*-value** |
| --- | --- | --- | --- | --- |
| Auditory (*F*(3, 223) = 3.53, *p* = .020, *R^2^* = .045) | | | | |
| **Intercept** | **0.29** | **0.13** | **2.19** | **.030*** |
| Segregation | -0.15 | 0.17 | -0.90 | .370 |
| Age (linear) | -0.07 | 0.07 | -0.95 | .345 |
| **Age (quadratic)** | **-0.24** | **0.09** | **-2.71** | **.007**** |
| CO (*F*(3, 223) = 3.34, *p* = .020, *R^2^* = .043 | | | | |
| Intercept | 0.30 | 0.19 | 1.57 | .118 |
| Segregation | 0.06 | 0.12 | 0.49 | .627 |
| Age (linear) | -0.03 | 0.08 | -0.38 | .704 |
| **Age (quadratic)** | **-0.23** | **0.09** | **-2.63** | **.009**** |
| FPN (*F*(3, 223) = 4.55, *p* = .013, *R^2^* = .058) | | | | |
| **Intercept** | **0.29** | **0.11** | **2.56** | **.011*** |
| Segregation | 0.23 | 0.12 | 1.93 | .055+ |
| Age (linear) | -0.02 | 0.07 | -0.30 | .765 |
| **Age (quadratic)** | **-0.23** | **0.09** | **-2.73** | **.007**** |
| MTL (*F*(3, 223) = 3.43, *p* = .020, *R^2^* = .044) | | | | |
| Intercept | 0.18 | 0.12 | 1.55 | .123 |
| Segregation | 0.06 | 0.09 | 0.72 | .471 |
| Age (linear) | -0.04 | 0.07 | -0.55 | .586 |
| **Age (quadratic)** | **-0.23** | **0.09** | **-2.66** | **.008**** |
| PM (*F*(3, 223) = 3.43, *p* = .020, *R^2^* = .044) | | | | |
| Intercept | 0.14 | 0.16 | 0.84 | .400 |
| Segregation | 0.10 | 0.14 | 0.72 | .471 |
| Age (linear) | -0.03 | 0.08 | -0.34 | .736 |
| **Age (quadratic)** | **-0.23** | **0.09** | **-2.63** | **.009**** |
| Reward (*F*(3, 223) = 3.97, *p* = .020, *R^2^* = .051) | | | | |
| Intercept | 0.11 | 0.13 | 0.78 | .435 |
| Segregation | -0.09 | 0.06 | -1.44 | .151 |
| Age (linear) | -0.07 | 0.07 | -0.97 | .335 |
| **Age (quadratic)** | **-0.23** | **0.09** | **-2.65** | **.009**** |
| SAL (*F*(3, 223) = 3.83, *p* = .020, *R^2^* = .049) | | | | |
| Intercept | 0.19 | 0.11 | 1.71 | .089+ |
| Segregation | -0.16 | 0.12 | -1.29 | .200 |
| Age (linear) | -0.10 | 0.08 | -1.22 | .223 |
| **Age (quadratic)** | **-0.22** | **0.09** | **-2.53** | **.012*** |
| Somatomotor Lateral (*F*(3, 223) = 3.35, *p* = .020, *R^2^* = .043) | | | | |
| **Intercept** | **0.22** | **0.11** | **2.08** | **.039*** |
| Segregation | 0.05 | 0.11 | 0.52 | .605 |
| Age (linear) | -0.03 | 0.08 | -0.32 | .750 |
| **Age (quadratic)** | **-0.23** | **0.09** | **-2.64** | **.009**** |
| VAN (*F*(3, 223) = 3.79, *p* = .020, *R^2^* = .049) | | | | |
| Intercept | 0.12 | 0.13 | 0.92 | .359 |
| Segregation | -0.11 | 0.09 | -1.25 | .214 |
| Age (linear) | -0.08 | 0.08 | -1.11 | .268 |
| **Age (quadratic)** | **-0.22** | **0.09** | **-2.52** | **.013*** |
| Visual (*F*(3, 223) = 3.41, *p* = .020, *R^2^* = .044) | | | | |
| Intercept | 0.07 | 0.25 | 0.30 | .764 |
| Segregation | 0.12 | 0.18 | 0.68 | .501 |
| Age (linear) | -0.04 | 0.07 | -0.54 | .589 |
| **Age (quadratic)** | **-0.23** | **0.09** | **-2.65** | **.009**** |

Overall model *p*-values are FDR-corrected. Bold font indicates a significant effect.

***p* < .01, **p* < .05, ^+^*p* < .10

**Table S3**. Cluster information for the task-based Decrease vs. Look Negative contrast.

| **Cluster** | **Size (voxels)** | **x** | **y** | **z** | **Location** |
| --- | --- | --- | --- | --- | --- |
| Decrease > Look Negative | | | | | |
| 1 | 11376 | 6 | -18 | 18 | Bilateral MTG, MFG, SFG, superior medial gyrus, IFG |
| 2 | 2108 | -13 | 75 | -24 | Cerebellum (bilateral Crus I, L Crus II) |
| 3 | 1214 | 45 | 63 | 30 | L angular gyrus, MTG, IPL |
| 4 | 799 | 2 | 52 | 18 | Bilateral precuneus, L calcarine gyrus, L PCC |
| 5 | 759 | -47 | 61 | 25 | R angular gyrus, MTG |
| 6 | 177 | 33 | -34 | 31 | L MFG |
| 7 | 157 | 11 | 84 | 20 | L cuneus, superior occipital gyrus |
| 8 | 101 | 3 | 45 | -37 | Bilateral cerebellum (IX) |
| 9 | 89 | 35 | 49 | -1 | L ITG, MOG |
| Look Negative > Decrease | | | | | |
| 1 | 7948 | 29 | 20 | 36 | L postcentral gyrus, precentral gyrus, STG, IPL, insula |
| 2 | 4629 | -47 | 16 | 21 | R STG, supramarginal gyrus, precentral gyrus, postcentral gyrus |
| 3 | 1044 | -15 | 66 | -1 | R Lingual Gyrus, cerebellum (VI), cuneus |

Coordinates refer to the center of mass in Talairach atlas space (RAI). IFG = inferior frontal gyrus; IPL = inferior parietal lobule; ITG = inferior temporal gyrus; MFG = middle frontal gyrus; MOG = middle occipital gyrus; MTG = middle temporal gyrus; PCC = posterior cingulate cortex; SFG = superior frontal gyrus.
